# Translational design of multifunctional novasomes for theranostic-driven precision therapy of cutaneous oncology

**DOI:** 10.1186/s13036-026-00744-8

**Published:** 2026-08-05

**Authors:** Md. Azhar, Rishabha Malviya, Phool Chandra, Sathvik Belagodu Sridhar, Javedh Shareef, Tarun Wadhwa, Daniel Arockiam

**Affiliations:** 1 Department of Pharmacy, School of Medical and Allied Sciences, Galgotias University, Greater Noida, UP 201308 India; 2 Teerthanker Mahaveer College of Pharmacy, Teerthanker Mahaveer University, Moradabad, UP 244001 India; 3 College of Pharmacy, Dubai Medical University, Dubai, United Arab Emirates; 4 RAK College of Pharmacy, RAK Medical & Health Sciences University, Ras Al Khaimah, United Arab Emirates; 5 School of Engineering and IT, Manipal Academy of Higher Education, Dubai Campus, Dubai, United Arab Emirates

**Keywords:** Novasomes, Nanovesicles, Intralesional, Cutaneous oncology, Theranostics, Localized

## Abstract

Cutaneous oncology needs to be treated locally with maximal exposure to the target with minimum systemic toxicity. Novasomes, surfactant-rich vesicles, have proven to be promising theranostic carriers for both topical and intralesional delivery. This review aims to establish a translational framework linking novasome design with theranostic-guided dosing in cutaneous oncology. A literature search of publications from the time period 2010–2026 was conducted in Scopus, PubMed, and Web of Science to capture studies on formulation, in vitro/ex vivo penetration data, in vivo efficacy, and biodistribution, and the first clinical reports; the priority was given to studies with orthogonal imaging, microdialysis, and/ or IND-relevant endpoints. The literature shows that novasomes allow improved local drug retention, controllable release kinetics, and better follicular and tumor penetration than traditional topical systems. Surface modification and deformable bilayers also help further optimise targeting, and co-loading imaging agents enables real-time visualisation, image-guided dosing, and adaptive PK/PD modelling. Advances in scalable fabrication techniques such as microfluidics and solvent-minimized processes enhance translational feasibility. However, there are still challenges in long-term stability, standardized reporting of critical quality attributes, and regulatory harmonization. In conclusion, novasomes are a pragmatic and modular platform for localized image-guided cutaneous oncology. A unique aspect of this review is the inclusion of novasome formulation strategies, theranostic functions, and concepts of PK/PD-guided dosing in a translational context for cutaneous oncology. The review bridges the gap between formulation development and clinical implementation aspects, thereby offering a practical view for future precision nanomedicine strategies.

**Graphical Abstract:**

**Figure:**
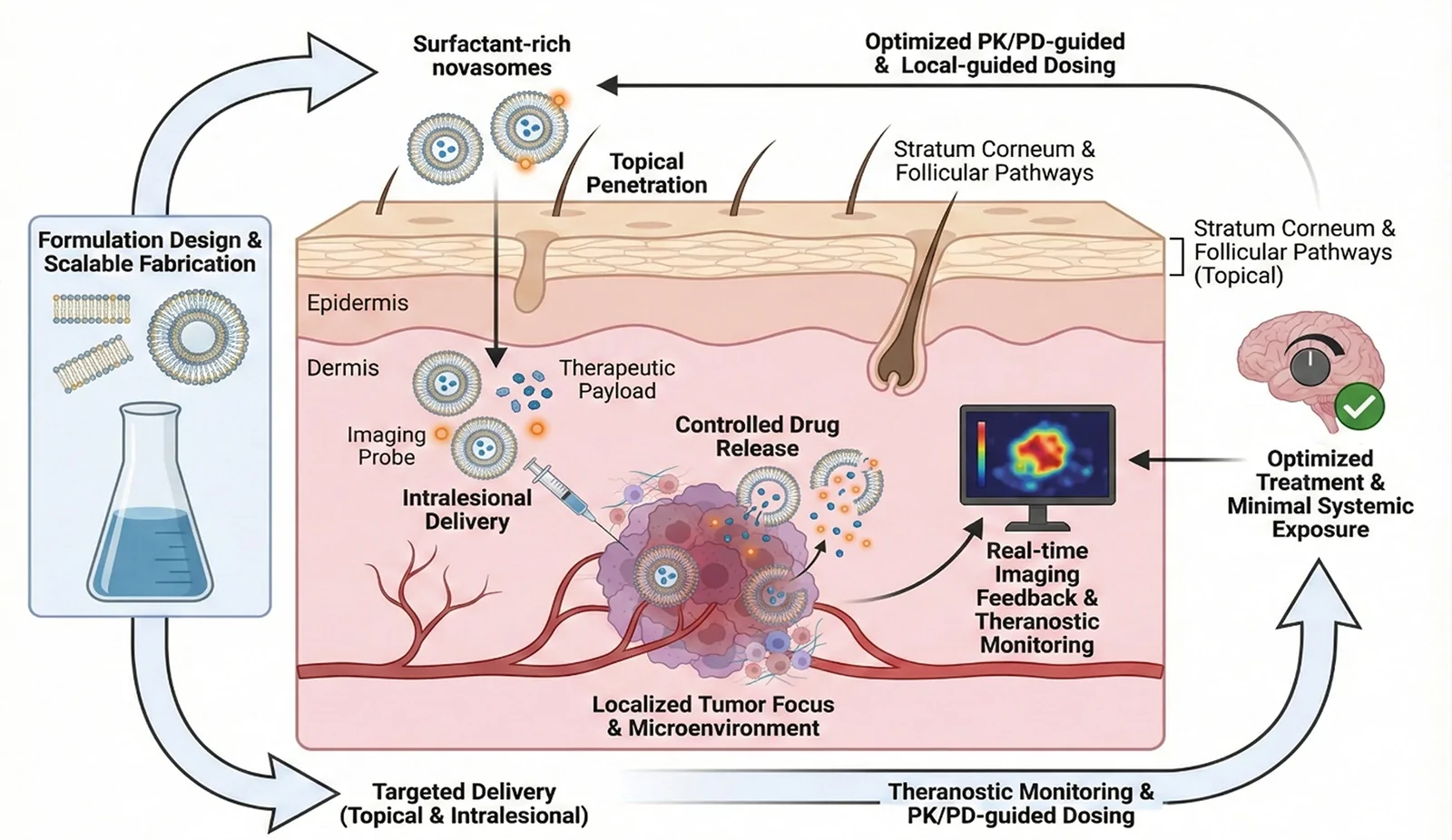

## Introduction

Skin cancer represents a major global health burden with significant biomedical and public health implications. Global cancer surveillance estimated close to 19.3 million new cancer cases in 2020 and reported a significant contribution of skin malignancy to the overall number of incidences in many parts of the world [1]. Maintained global case numbers at a high level, updated estimates for 2022 [2], and confirmed geographical differences in occurrence and mortality, reflecting variation in the environment, demographic, and health-care systems. Non-melanoma skin cancers (NMSCs), which are mostly basal cell carcinoma and squamous cell carcinoma, are the most common reason for a new diagnosis of a cutaneous tumor. Melanoma has a lower occurrence and a relatively large death rate, and the capacity to spread to other parts of the body. The morbidity in terms of skin cancers includes deformity, loss of function due to cancer, repeated surgical intervention, and long-term surveillance requirements. Standard management of localized disease is based on surgical excision and (selective) radiotherapy [3].

The skin barrier is a significant challenge for local drug delivery. Multiple therapeutic molecules are limited in passive diffusion through the stratum corneum. The distribution of drugs in the tumor tissue is also restricted due to heterogeneous perfusion and the complex microenvironment of the tumor. Nanocarrier technologies offer opportunities to enhance local bioavailability while reducing off-target exposure. Nano-vesicular systems allow drug solubilization, degradation protection, and controlled drug release, and modified interactions with skin appendages such as hair follicles as reservoirs for particle uptake [4, 5]. Lipid-based nanocarriers in particular have translational promise due to favorable biocompatibility, scalability of manufacture, and precedence of regulation for related systems [5, 6]. The modular form of nanocarriers offers the possibility of the delivery of multiple payloads at the same time. This property is in favor of combination therapy and the combination of diagnostic labels for therapeutic functions [4–6].

Novasomes fill a pragmatic gap belonging to the group of vesicular systems for topically and intralesionally active applications. These multi-bilayer vesicles containing surfactant-rich matrices are capable of transporting both hydrophilic and hydrophobic cargoes at fairly high loadings. Formulation flexibility allows the use of edge activators, fatty acids, and sterol components, optimizing the fluidity and skin interaction of the bilayer. Novasomal architectures can be modified for advanced targeting strategies including ligand-mediated and stimuli-responsive approaches have been investigated and are discussed in detail in section “Targeting strategies and skin/tumor penetration”. Published formulation and characterization studies show that novasome platforms improve penetration, raise the local retention, and decrease systemic exposure in preclinical models and exploratory human assessments [7, 8]. These characteristics are favorable for translational interest in the development of novasomes for the treatment of cutaneous oncology, particularly for anatomically challenging or non-surgical desires of patients.

The diagnostic integration of nanosystems provides one additional benefit for cutaneous oncology. Co-loading of imaging agents within nanovesicles using imaging techniques enables imaging of the drug distribution and noninvasive monitoring of treatment distribution and image-guided change of dosing schedules. Fluorescent probes, magnetic particles, and radionuclide chelates for fluorescence imaging, MRI mapping, or PET imaging have been employed in nanocarrier platforms, respectively, to allow preclinical imaging. Theranostic designs support response-guided dosing and might eliminate the number of invasive biopsies needed for clinical decision making [9, 10]. Combining diagnostic readouts with therapeutic payloads is in line with modern aspirations for precision oncology and can help to expedite early-phase clinical evaluation of local pharmacokinetics and pharmacodynamics.

This review is a focused and translationally-oriented framework for the development of multifunctional novasome formulations for cutaneous oncology. The novelty of this review is that it brings the formulation design, theranostic imaging strategies and consideration of PK/PD-guided dosing all together in a single translational framework for cutaneous oncology. Unlike the previous reviews that have mainly concentrated on nanovesicular carriers or clinical translation pathway/SKIN nanomedicine, the present one emphasizes the novel connection of novasome engineering with image-guided monitoring, localized dosing strategies and clinical translation pathway. This holistic view could help further the evolution of precision nanomedicine strategies for localized skin cancers. The various objectives are providing a clear synthesis of the current state of the art of novasome composition and variants, providing an appraisal of fabrication and scale-up strategies, and providing a practical discussion of physicochemical and biological characterization methods. The targeting and penetration strategies relevant to skin tumor types in this context are also collected, and the options for combining diagnostic payloads with skin tumor types, as well as for designing dosing regimens with local efficacy and systemic safety at the forefront, are also highlighted. Preclinical models and recommended IND-enabling endpoints receive special attention to facilitate strong translational pathways. Comparative insights are provided to put novasomes in a larger context of nano-delivery, alongside other lipid vesicles and topical nano-platforms [5, 6, 11].

## Novasome fundamentals: composition, structure, and variants

Novasomes are an Advanced class of vesicles carrier constructed using nonionic surfactants together with complementary lipids. The term defines engineered, bilayered vesicles, which incorporate the membrane formation driven by surfactants with cholesterol or other stabilizers to produce a powerful, deformable vesicle. The development of novasomes originated from earlier research on surfactant-based vesicles and lipidic carriers and matured during the 2000s and 2010s [7, 8]. According to early reports, non-ionic surfactants were found to have great value in stable bilayer formation as well as economical production, as opposed to pure phospholipid systems [12]. The Novosome ideas evolved to controlled encapsulation of hydrophobic drugs, where investigators proved to co-load imaging moieties, which were later usable in theranostic applications [7, 8].

At their core, novasomes consist of surfactant-rich bilayers stabilized by cholesterol or other sterol derivatives. Common surfactants are classes of span and tween and advanced, customized nonionic amphiphiles. Cholesterol controls membrane packing as well as rigidity. When enhanced biocompatibility or definite mechanical properties are needed, helper lipids and neutral phospholipids are also included [6, 13]. The polar solvents, buffered salts, or polymeric hydration agents are present in hydration media to adjust inner aqueous phase properties. Single-chain surfactants, or short-chain alcohols, are added to increase vesicle deformability and skin penetration to be used topically [14]. These choices of composition establish the level of stability, loading, and performance.

Several functional variants of novasomes have been developed to address specific therapeutic and diagnostic requirements. Classic novasomes adhere to an uncomplicated surfactant-cholesterol-hydration equation and are adjusted to deliver optimal topical loads and follicular deposition [7, 15]. PEGylated novasomes are the novasomes that have surface-grafted polyethylene glycol chains. PEGylation decreases the adsorption of proteins and increases the colloidal stability of systemic or intralesional biomedical applications [6, 16]. The decoration of ligands onto novasomes is done with peptides, aptamers, or small-molecule ligands at regulated densities. Ligand display facilitates active targeting to overexpressed receptors on tumor or stromal cells, and enhanced cellular uptake and retention can be enhanced [16, 17]. Stimulus-responsive shapes incorporate pH-responsive lipids, thermo-responsive blocks, or enzyme-cleavable linkers to cause cargo release in the acidic or protease-rich microenvironment of the tumor [15, 18]. Theranostic-loaded novasomes include therapeutic drugs and imaging probes, e.g., fluorescent dyes, MRI chelates, or radionuclide tags in their theranostic-loaded novasomes and are delivered and monitored concurrently [8, 10].

The performance of novasomal batches is characterized by key physicochemical measures, which define the subsequent performance. The reported hydrodynamic size and particle size distribution in terms of mean diameter and PDI control the tissue penetration, follicular deposition, and the clearance behavior. Zeta potential is used to determine and inform the colloidal stability and charge-based interaction with skin and cells. Lamellarity and bilayer thickness have an impact on loading capacity and release kinetics. The percentage of loading capacity (EE%), as well as encapsulation efficiency (EE%), measures the proportion of active inside vesicles to free drug. DSC, fluorescence anisotropy, or micropipette is a measure of bilayer rigidity and predicts release profile and deformability across the stratum corneum. Collectively, the parameters can be used to determine the choice of formulations to be used in topical or intralesional administration and should always be reported to ensure comparability [6, 12, 19].

The analysis of the characterization of novasomes is based on a familiar toolkit. The mean size and PDI are given through dynamic light scattering. Zeta potential is provided by electrophoretic mobility. It can be seen by electron microscopy (TEM/ cryo-TEM) and AFM. Bilayer order and drug-excipient interaction are investigated by thermal and spectroscopic methods (DSC, FTIR, SAXS). The amount and stability of drugs are measured chromatographically. The sink conditions and Franz diffusion cells release testing are essential in order to predict departures of topical systems in the skin. To measure imaging payload integrity and signal stability, relaxometry, fluorescence imaging, or radiometric assays are used to measure theranostic designs [3, 6, 8]. The combination of these techniques defines CQAs in novel vesicles of preclinical and regulatory packages.

Novasomes possess a number of formulation assets in cutaneous and intralesional oncology. Their lipophilic anticancer agents can be highly loaded using their bilayers that contain high concentrations of surfactants. The composition of the membrane can be adjusted to achieve burst and sustained release so that local high exposures and systemic low spillover may be achieved. Surface functionalization and deformable vesicle design may enhance tissue targeting and penetration. These targeting mechanisms are discussed in detail in section “Targeting strategies and skin/tumor penetration” [7, 15]. The ability to accommodate imaging payloads further expands the functional versatility of novasomes, with theranostic applications discussed in later sections (Section “Diagnostic and theranostic integrations”). It is a modular composition that is compatible with several types of administration, such as creams, gels, injectables, and microneedle reservoirs [1, 9, 10].

Novasomes have a role that can be understood by comparing them to related vesicular carriers. Liposomes are phospholipid and are also excelling in biocompatibility and clinical pedigree. Non-ionic surfactants allow novasomes to exchange a portion of the pure phospholipid nature with strength and cheaper cost, whilst maintaining the encapsulation properties of the bilayer [12]. Niosomes have an identical non-ionic platform with novasomes, yet, as part of a distinct surfactant chemistry and manufacturing corporate culture, niosomes are highlighted as being stable and low-cost, whereas novasomes are designed to be loaded with therapeutic payloads and theranostically prepared in cutaneous conditions [20]. Ethosomes are high ethanol and flexible bilayers that are designed to penetrate the skin at depths using deep targeting; novasomes are designed to offer deformability as well as engineered pockets and targeting motifs instead of using ethanol alone to enhance permeability [20, 21]. These comparisons place novasomes as a hybrid solution that is localized to satisfy the oncology requirements.

The feasible strengths of novasomes are manufacturing and formulation control. Novasomes are easily adapted to many of the preparation paths that have been developed with vesicles, such as thin-film hydration, ethanol injection, and microfluidic mixing. Surfactant: cholesterol ratio, hydration volume, and post-processing (sonication/extrusion) are all process variables that can be predictably tuned to determine size and EE. Scale-up needs shear, solvent removal, and sterile manipulation to injectable or intralesional format, however, the relative chemical stability of surfactant materials simplifies sourcing of raw materials and storage relative to labile phospholipids [6, 12]. The advantages of regulatory translation include definite CQAs and theranostic traceability in case imaging labels are of GMP-grade [6, 18].

However, despite the several interesting physicochemical properties, many of the proposed benefits of novasomes are still largely supported by preclinical data, and direct comparative studies are often hindered by the different compositions of the formulations, methods of characterization, and models of biological evaluation used. While some benefits are proposed such as the ability to load more drug, or the flexibility of formulation, many others are probably limited by the increased complexity of the formulation and the greater batch to batch variability compared to liposomes. In addition, clinical validation of novasomes is still limited, and the number of clinical and regulatory experiences is far less than that in liposomes.

**Fig. 1:**
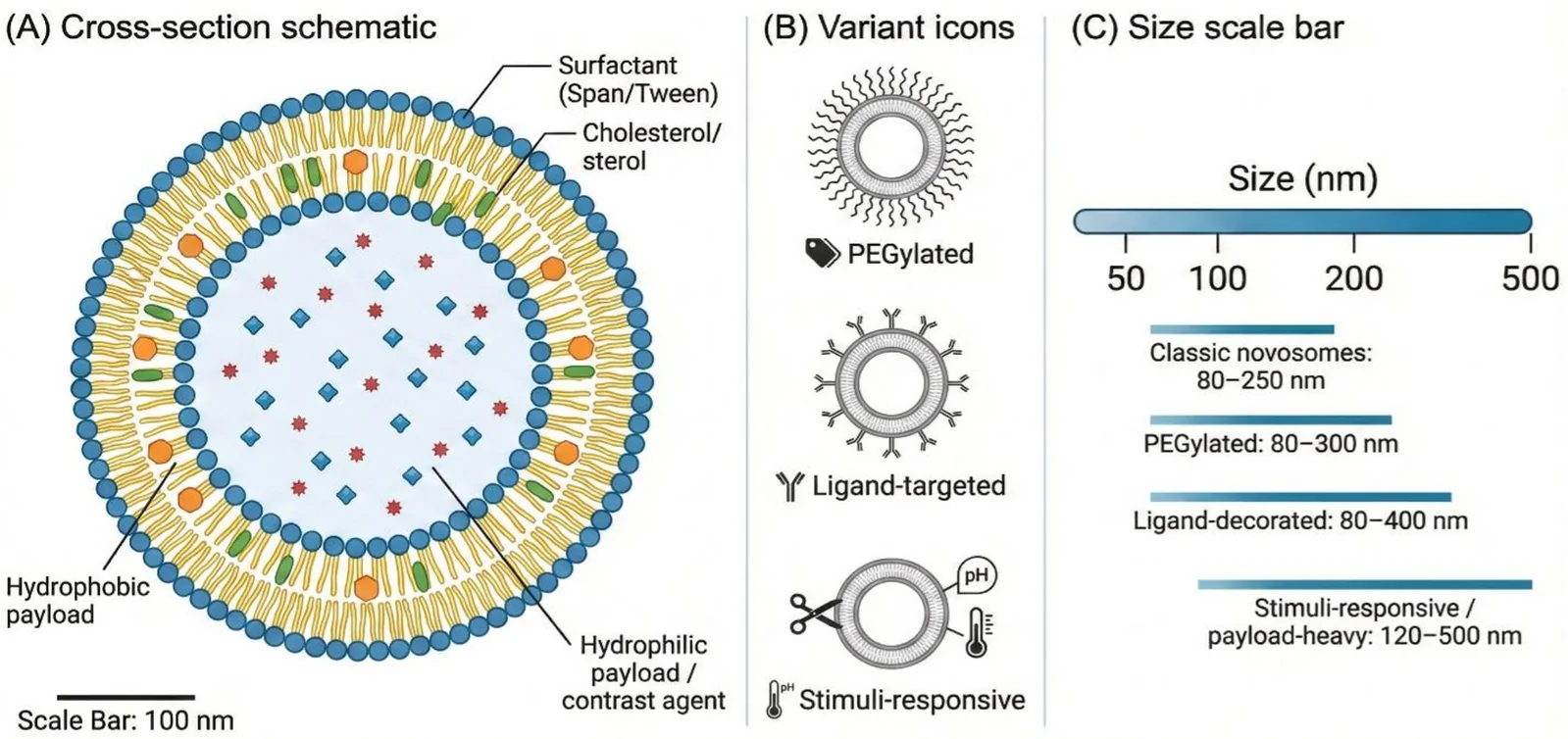
Structural architecture of novasomes and major functional modifications, including PEGylation, ligand decoration, stimulus-responsive design, and theranostic integration for cutaneous oncology applications

Overall, novasomes are formed by the association of surfactant bilayers with sterol stabilizers and modular surface chemistry to provide a flexible platform. Figure 1 shows the simple structure of novasomes and points out the main structural modification used to produce the functional molecules for application to cutaneous oncology. Topical and intralesional oncology application is compatible with the platform due to its ability to load high lipophilic drugs, release them controllably, deform to penetrate the skin, and be easily coupled with imaging agents. To have reproducible research, careful physicochemical characterization and standard reporting will be required, as well as to move the clinical translation forward. The following sections of this review will discuss fabrication, characterization, targeting strategies, theranostic integration approaches, and dosing approaches in more detail. Table 1 presents a comparative overview of some widely used nano-system platforms.

**Table 1:** Mechanistic, formulation, and translational comparison of major nanosystem platforms used for drug delivery in cutaneous oncology

System	Size (nm)	Mechanistic features	Key strengths	Main limitations	Translational relevance / applications	Ref.
Novasome	80–400	Surfactant-rich bilayer with high deformability; supports ligand decoration, PEGylation, and co-loading of therapeutic and imaging agents	High lipophilic drug loading; modular design; good follicular entry; suitable for theranostic integration	Batch variability if process is not optimized; fewer clinical/regulatory precedents than liposomes	Topical and intralesional oncology; image-guided local therapy; localized precision delivery	[7, 8, 22]
Liposome	50–200	Phospholipid bilayer closely resembles biological membranes; widely used for passive and active encapsulation	High biocompatibility; extensive clinical history; strong regulatory familiarity	Higher cost; phospholipid oxidation/hydrolysis; limited loading for some highly lipophilic drugs	Systemic and topical delivery; vaccine carriers; clinically established drug encapsulation platform	[12, 23]
Niosome	80–300	Surfactant bilayer with cholesterol stabilization; chemically more stable than phospholipid-based systems	Cost-effective; good colloidal stability; suitable for topical use	Less clinical precedent; performance depends strongly on surfactant choice	Topical delivery; transdermal patches; dermatologic drug delivery	[12, 20, 24]
Ethosome	50–300	Ethanol increases bilayer fluidity and skin permeation, improving dermal transport	Strong penetration enhancement; useful for deep skin delivery	High ethanol may irritate sensitive skin; limited suitability for some payloads	Transdermal and deep dermal delivery of small molecules and peptides	[21, 25]
Solid Lipid Nanoparticle (SLN)	50–300	Solid lipid matrix controls diffusion and release of drug	Good physical stability; scalable production; controlled release	Limited drug loading; polymorphism and drug expulsion during storage	Topical formulations; oral/parenteral controlled release	[6, 26]
Nanostructured Lipid Carrier (NLC)	80–400	Imperfect lipid matrix improves drug accommodation and reduces crystallization	Improved loading compared with SLN; tunable release; better storage performance	Formulation optimization can be complex; stability depends on lipid composition	Topical and parenteral controlled release; dermal targeting	[6, 26]
Polymeric nanoparticles (e.g., PLGA)	50–300	Polymer degradation enables sustained release; surface can be functionalized for targeting	Controlled degradation; sustained release; versatile surface engineering	Possible polymer/metabolite issues; more complex regulatory pathway	Sustained release injections; targeted delivery; vaccine platforms	[27]
Micelles (polymeric/ surfactant)	10–100	Hydrophobic core solubilizes poorly soluble drugs; small size favors diffusion	Excellent solubilization of hydrophobic drugs; small size may aid penetration	Less stable on dilution; mainly suitable for lipophilic payloads	Solubilization of poorly soluble drugs; topical formulations; parenteral solubilizers	[27, 28]

Although novasomes, niosomes, and liposomes all belong to the broader class of vesicular nanocarriers, important mechanistic and translational differences exist among them. Liposomes are made up of phospholipid bilayers, and are highly biocompatible and well accepted in clinics because they closely mimic biological membranes. The disadvantages may be the phospholipid oxidation, the phospholipid hydrolysis and the relative high manufacturing costs, which could impact long-term stability and large-scale production [12]. Niosomes are made up of non-ionic surfactant and cholesterol and their chemical stability and production cost is better than liposomes. However, their drug loading capacity and the multifunctional engineering potential may be restricted depending on the composition of the surfactant [24]. The next step in evolution of surfactant-based vesicular systems are the Novasomes, which contain higher amounts of surfactant, multiple bilayers, and more flexibility in formulation. This architecture allows for higher loadings of lipophilic agents, addition of targeting ligands and embedding of imaging payloads for theranostic applications [8, 22]. With regard to the translation, liposomes are presently most well founded in terms of regulatory and clinical aspects, while niosomes and novasomes are mostly in preclinical stages. Nevertheless, the improved modularity and multifunctionality of novasomes could be beneficial for future precision oncology, if issues with the reproducibility of manufacturing, long-term stability, and clinical validation can be overcome.

## Fabrication and formulation strategies

Novasomes are prepared using principles similar to those employed for other lipid–surfactant vesicular systems, with modifications to accommodate non-ionic surfactants, edge activators, and dual-payload loading strategies. The thin-film hydration method is still the most widespread method of laboratory production. In the case of thin-film hydration, the lipid and surfactant components are dissolved in an organic solvent and dried to obtain a thin film. The film is aqueous hydrated to give multilamellar vesicles. Subsequent size reduction is achieved using sonication, extrusion, or high-pressure homogenization to obtain vesicles with the desired size distribution. This approach is simple and has a lot of flexibility, and it enables direct inclusion of hydrophobic actives into the lipid bilayer and hydrophilic actives into the aqueous phase using them during the hydration or remote loading steps. The thin-film route has some limitations with respect to scale-up due to handling of solvent in a batch and variation in mixing of different batches, but still, it is the baseline technique for formulation development and proof-of-concept studies [23].

Reverse-phase evaporation results in large unilamellar vesicles of relatively high aqueous encapsulation. In this method, lipids and surfactants are dissolved in an organic phase, which is emulsified with an aqueous phase containing a hydrophilic payload. The organic solvent is removed under reduced pressure to leave vesicles. Reverse-phase evaporation can raise the amount of entrapped aqueous volume, and therefore is advantageous in water-soluble imaging probes or hydrophilic drugs. The process requires careful removal of solvents and causes problems with the residual solvent limit for translational work. The method is of value for certain kinds of payloads that require high aqueous loading [29].

Ethanol injection and solvent displacement techniques are possibilities that provide a one-step, mild procedure to produce the formation of nanosized vesicles. Lipid and surfactants are dissolved in water-miscible, organic solvents like ethanol and injected into an aqueous solution (stirred) or controlled mixing. Rapid diffusion of the solvent is responsible for self-assembly and rather uniform vesicles. Ethanol-based methods readily translate to continuous flow as well as microfluidic implementations and allow relatively simple removal of organic solvent. These methods are attractive where gentle processing and continuous manufacture are required [30].

Microfluidics allows for the precise control of mixing and self-assembly, and decreases polydispersity. Continuous-flow hydrodynamic focusing and microchannel mixing enable control of the particle size through the variations of flow-rate ratios and lipid concentrations. Microfluidic platforms allow the reproducible, narrow particle size distributions as well as reduced post-processing. The hurdles to be overcome include fabricating the channels, initial equipment cost, and scale-out strategies. Scale-out by parallelization and high-throughput microfluidic designs can fill the gap between running in the lab and pilot production, and can do this without sacrificing the control over mixing offered, which produces uniform batches [31].

Continuous manufacturing allows for improving throughput and reproducibility, however, critical quality attributes of the lipid-based vesicular system can be altered with scale-up, including particle size, polydispersity, encapsulation efficiency, and vesicle integrity [32, 33]. The literature reveals that the flow conditions, mixing intensity and process design have a significant impact on the formation and performance of vesicles [34] and can be precisely controlled by microfluidic approaches, which are characterized by high throughput and size control. Similarly, one can expect that similar effects will occur with novasomes, in the case of dual-loaded theranostic formulations, for instance, these shear stresses and dilution history might influence the retention of payloads and the stability of the bilayers. Hence, it is advisable to design the scale-up based on QbD, while maintaining tight control over the CPPs, to ensure the structure and functional performance are maintained [32–34].

Post-formation size controlling is of critical importance. Sonication and extrusion are still the common methods used to achieve narrow size distributions and specific mean size diameters. Sonication is an application of acoustic energy to disintegrate larger multilamellar vesicles into smaller units. Extrusion allows the vesicles to be forced through defined pore membranes to impose upper size limits related to the membrane pore diameter. Extrusion provides tight control, but may be time-consuming and result in a loss or membrane fouling of the product at larger scales. High-pressure homogenization is an option for scale-up; however, it needs optimization for encapsulated payload integrity [31].

Some of the process variables that have a major impact on batch attributes are surfactant to lipid ratio, cholesterol content, hydration medium composition, temperature at formation/hydration, sonication energy/time, and extrusion pore size. Surfactant content modulates bilayer packing and permeability, and a higher surfactant fraction can enhance the fluidity and deformability of the membrane, therefore facilitating the penetration across a barrier in the case of cutaneous use (follicle/transdermal). Cholesterol has a dual role of acting as a bilayer modulator where moderate levels of cholesterol increase membrane stability and reduce leakage, whereas excessive levels of cholesterol may decrease encapsulation of certain hydrophobic actives and alter release kinetics. Ionic strength and pH of the hydration medium have an effect [35].

There are more strategies for encapsulation based on the polarity of the payload. Hydrophobic small molecules and lipophobic imaging probes partition in the bilayer during the steps of dissolving the lipids. Hydrophilic agents, dyes, and radionuclide chelates are entrapped in the aqueous core by incorporating them in the hydration phase or using remote loading approaches. Dual-loading of therapeutic plus diagnostic agents is possible by combinations of bilayer partitioning dyes or contrast agents and aqueous core drugs. Careful stoichiometry and order of addition must be used so as not to cause competitive partitioning and keep the system colloidal. Remote loading (e.g., ion gradient methods) can be used to increase the encapsulation efficiency for amphoteric drugs [36, 37].

Industrial scalability and manufacturing reproducibility remain important considerations for the successful clinical translation of novasomes. Laboratory-scale methods like thin-film hydration can be used for formulation development but industrial production would require robust, scalable, and GMP-compatible manufacturing processes. The application of Quality by Design (QbD) principles can help with systematic optimization by determining Critical Quality Attributes (CQAs) such as particle size, polydispersity index, zeta potential and encapsulation efficiency, and Critical Process Parameters (CPPs) that affect product performance. Continuous manufacturing technologies like microfluidics can help enhance process control and minimize inter-batch variability. Besides, following standard analytical methods, process validation and in-process quality monitoring is crucial for ensuring the reproducibility, product uniformity and regulatory compliance. Solving these manufacturing issues will be essential in bringing novasome formulations from laboratory development to commercial production [38].

**Fig. 2:**
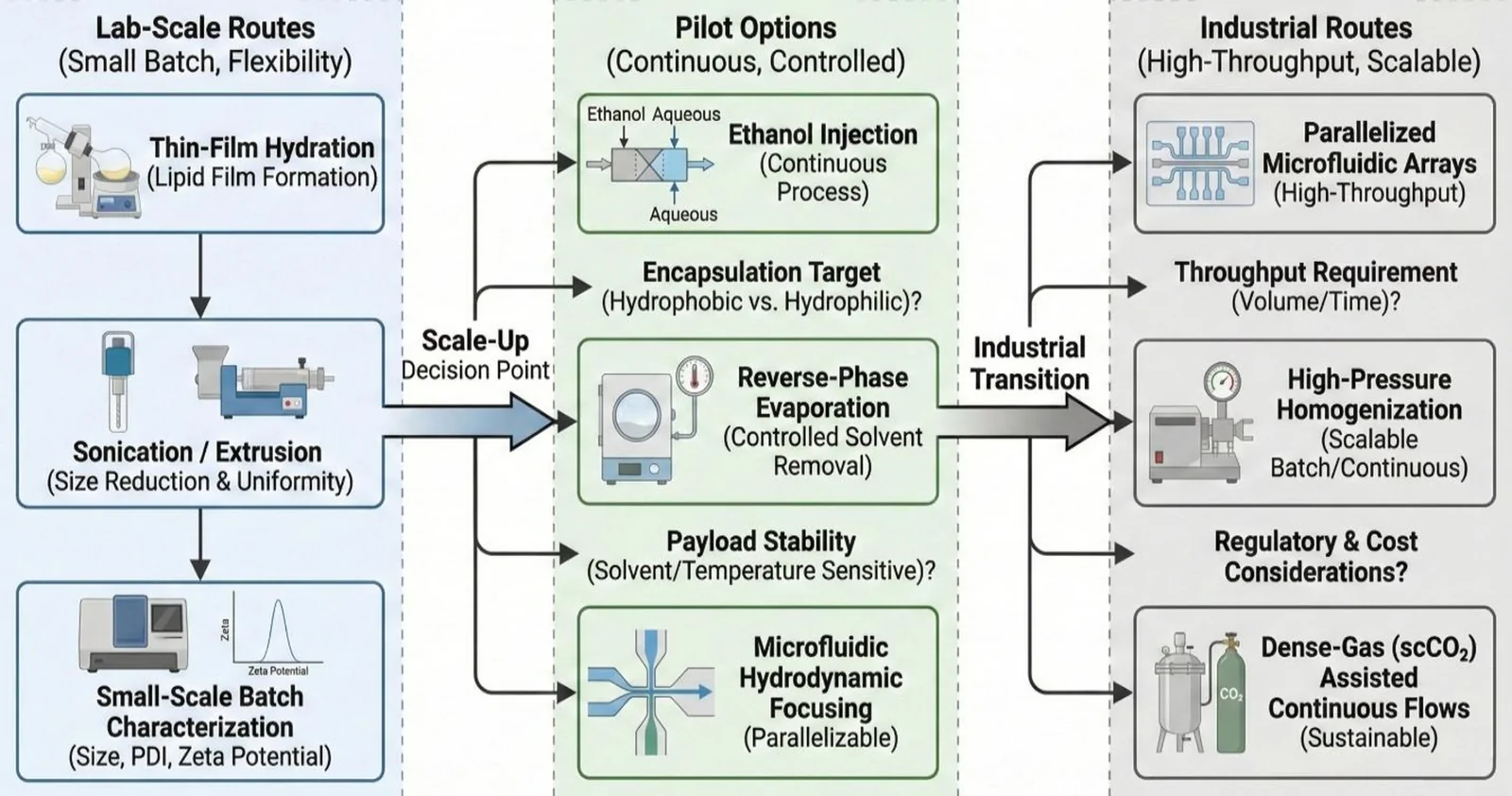
Overview of laboratory-scale, pilot-scale, and industrial-scale fabrication strategies for novasome manufacturing

Scale-up considerations require consideration of solvent handling, residual solvent limits, process reproducibility, and in-line quality controls. Microfluidic and continuous ethanol injection platforms scale by either parallelizing the operation or increasing throughput pumps, but not by increasing the reactor volume. Dense-gas or supercritical CO2-assisted methods offer solvent-minimizing solutions and low-hazardous environmental profiles, and may even facilitate solvent removal at the end of the process, and are promising candidates for industrial translation, but require process validation depending on the type of payload [39]. Figure 2 describes lab and industrial novasome fabrication workflows and compares the criteria of scalability, throughput, and batch reproducibility.

Sterility and aseptic processing are critical differences in the case of topical and intralesional formulation. Terminal sterilization may not be required for topical products that are applied to intact skin, but it must undergo microbial levels and preservative efficacy testing. Intralesional formulations are considered to be parenteral products and therefore need validated sterility assurance. Filtration of final nanosuspensions can be difficult if the particle size is close to the filter pore size. Aseptic manufacturing with sterile component inputs and sterile-to-sterile filling is often the strategy of choice with labile vesicular products. Conventional terminal sterilization techniques like heat or gamma irradiation are generally associated with damage to the vesicle integrity and the payload. Conventional approaches for terminal sterilization, such as heat or gamma irradiation, often damage the integrity of the vesicles and the payload; alternative sterilization techniques (sterile filtration, aseptic fill, or emergent technologies such as ScCO2) are recommended and have to be validated for each formulation [36, 40].

Analytical controls need to be incorporated in the process design. Typical tests performed during the release and in-process phase are particle size and PDI (DLS), zeta potential, encapsulation efficiency (HPLC/UPLC), residual solvent (GC), or sterility/mycoplasma tests. For theranostic formulations, specific analytical assays for imaging payload stability and functionality for the imaging of payload’s stability and action (e.g., relaxometry for MR-agents, radiochemical purity of PET/SPECT-labels) are required. Release testing strategies should reflect the intended dosage form and route, while set-up for topical release (Franz-type diffusion) or injected suspensions (dialysis or buffer release model) is standard. To improve interstudy comparability [29, 39, 41], Kinetic modelling and compliance under sink conditions are improved.

Although considerable efforts have been dedicated to developing fabrication technologies, several challenges related to the translation of these methods to industrial production have yet to be addressed: Laboratory-scale technologies like thin-film hydration and sonication are very flexible during formulation development, but often suffer from reproducibility issues at a larger scale.

In summary, a pragmatic fabrication strategy for novosomes involves iterating through formulation techniques (lab-scale thin-film/ethanol injection), followed by controlled size reduction (extrusion/sonication), and then microfluidic or continuous approaches (scale translation). Process variables need to be correlated with critical quality attributes as early as the development. Appropriate analytical assays and methodology for planning for sterilization also need to be incorporated into the process design for intralesional products. The choice of manufacturing method represents a balance between the desired physicochemical properties, encapsulation requirements, payload stability, and scalability [7, 22, 29, 30, 39, 42, 43]. Table 2 summarizes major novasome fabrication methods, key parameters, and scalability and formulation limitations relevant to cutaneous applications.

**Table 2:** Comparative evaluation of major novasome fabrication methods, process parameters, formulation characteristics, scalability potential, and associated limitations

Method	Key parameters	Stability and biocompatibility	Encapsulation efficiency	Limitation	Ref.
Thin-film hydration	Surfactant-to-lipid ratio; cholesterol content; hydration medium; hydration temperature; post-sonication/extrusion	Good physical stability; high biocompatibility due to non-ionic surfactants; suitable for topical use	Moderate to high (40–95%) depending on payload	Batch-wise process; solvent removal; limited industrial scalability	[13, 22, 29]
Reverse-phase evaporation	Organic/aqueous phase ratio; emulsification energy; solvent removal rate	Stable vesicles; acceptable biocompatibility after solvent removal	High for hydrophilic payloads	Residual solvent risk; complex processing	[22, 44]
Ethanol injection	Lipid concentration; injection rate; mixing speed; temperature	Good stability; biocompatible if residual ethanol is minimized	Moderate	Organic solvent handling; limited control for very large batches	[22, 30]
Microfluidics (flow-focusing)	Flow-rate ratio; channel geometry; lipid concentration	Excellent colloidal stability; high biocompatibility; reproducible batches	Moderate to high	High initial equipment cost; scale-out required	[22, 35, 42]
Sonication (size-reduction)	Sonication energy; duration; temperature control	Acceptable stability; risk of payload degradation if overheated	Not applicable (post-formation step)	Possible drug degradation; metal contamination	[22]
Extrusion	Membrane pore size; number of cycles; pressure	High stability and uniformity; biocompatible	Not applicable (post-formation step)	Membrane clogging; material loss	[22]
Supercritical CO₂-assisted methods	Pressure; temperature; lipid solubility in scCO₂	Excellent biocompatibility; solvent-free residues	Variable	Process complexity; limited industrial adoption	[22, 39]

## Physicochemical characterization and analytical methods

Physicochemical characterization forms the foundation for evaluating the quality, stability, and biological performance of novasome formulations. Particle sizing and polydispersity index (PDI) are very important for characterizing. PDI is a degree of particle size distribution uniformity, with values below 0.2 being monodisperse novasomes, 0.2–0.3 being acceptable, while values above 0.3 suggest broad unstable size distribution [41]. Dynamic light scattering (DLS) provides hydrodynamic diameter and PDI and is currently the standard apparatus for checking the quality of nanosuspensions. DLS analysis should include reporting of number-, volume-, and intensity-based distributions, with appropriate sample dilution to minimize multiple scattering and aggregation artefacts. As well as being able to report scattering angle and temperature, regular instrument calibration and reporting make comparisons between studies more comparable [41, 45].

Surface charge evaluation using zeta potential measurement provides information on colloidal stability and propensity for interaction with biological media. Electrophoretic mobility measurements converted to zeta potential must include information on medium ionic strength, pH, and the applied model for data inversion. Zeta potential is the electrical potential in the slipping plane of the dispersed particle that is indicative of colloidal stability. Higher absolute values indicate higher electrostatic forces and less clumping. For novasomes, ≥ ±25 mV indicates good stability, whereas near neutral values are indicative of steric stabilization mechanisms [46, 47]. Consistent reporting of measurement conditions is helpful for the topical and intralesional applications in increasing their interpretability. High-resolution imaging complements ensemble techniques by providing direct visualization of vesicle morphology and lamellarity. Cryo-transmission electron microscopy (cryo-TEM) is a way to obtain structural snapshots of vesicles near native conditions, not to mention the fact that multilamellar architecture and the encapsulation of the cargo are also preserved. An inclusion of representative micrographs with scale bars and particle size overlays is recommended regularly to make microscopy data relate to DLS data. Standardized vitrification and low-dose imaging - incorporating a minimization of artefacts and damage to the radiation beam [48, 49].

Thermal and structural analyses provide information on bilayer organization, membrane order, and drug-excipient interactions. Differential scanning calorimetry (DSC) reports phase transition temperature & enthalpy indications of bilayer packing, sterol & payload incorporation. Fourier-transform infrared spectroscopy (FTIR) is used to detect the changes in lipid and drug vibrational bands due to the occurrence of hydrogen bonds or other interactions. Small-angle X-ray scattering (SAXS) and wide-angle scattering are used to quantify the thickness of bilayers, repeat spacing, and lamellarity at a statistically representative volume. These orthogonal methods characterize membrane rigidity and predict the release behavior altogether [50, 51].

The rheological properties of novasomes are very relevant if they are added to semi-solid topical formulations like gels and creams. Formulation parameters such as viscosity, storage (G′) and loss (G″) moduli, and thixotropy affect formulation stability, spreadability, and skin residence time. The appropriate viscoelastic properties will ensure that the vesicles are well distributed in the formulation during storage and will minimize the chances of aggregation or phase separation. Furthermore, the rheological behavior can also play a direct role on the diffusion of drugs and drug release kinetics by affecting the mobility of the encapsulated vesicles in the semi-solid matrix. The selection of a highly viscous or a lower viscosity system may be the determining factor for increased residence time and sustained drug release (highly viscous) versus more rapid drug release and skin penetration (lower viscosity). Thus, rheological tests should be considered as useful complementary tests for achieving a stable topical novasome formulation with optimum therapeutic performance [52, 53].

Quantitative determination of drug content and validation of the assay are important for the determination of encapsulation efficiency and the report of release. The chromophoric or UV-absorbing drugs are used as the primary method of quantification by HPLC or UPLC methods. Method validation parameters include specificity, linearity, accuracy, precision, LOD/LOQ, and stability, which indicate the capability. LC-MS or LC-MS/MS provides an even higher level of selectivity and sensitivity when used for bioanalysis and samples with complex matrices such as skin extracts or plasma. Clear documentation of extraction recovery and matrix effects is required in PK and tissue distribution studies [54, 55].

Route and dosage form of in vitro release testing should be indicative of intended use. Franz diffusion cells are still the accepted standard in the testing of topical and semi-solid formulations because it allows for controlled donor and recipient compartments and also allow for the measurement of permeations across the membranes or the skin. For suspension-based injectables or intralesional formulations, dialysis or sample-and-separate buffer release systems are available, which give cumulative release profiles. Sink conditions, sufficient sampling frequency, and reporting of cumulative percent release with standard deviations are needed for the use of kinetic modelling. Model fitting against zero-order, first-order, Higuchi, and Korsmeyer-Peppas forms argues in favor of a mechanistic interpretation and comparison between formulations [56, 57].

Stability testing is guided by stability paradigms adopted in nano systems. Short-term, accelerated, and long-term studies, as per guidelines in ICH, establish shelf life and storage conditions. Photostability testing is needed with light-sensitive payloads and formulations that are to be used topically. Some of the stability endpoints that are tested are particle size/PDI, zeta potential, encapsulation efficiency, residual solvent, potency assays, etc. Documentation of protocol details of temperature, humidity, and light exposure are indispensable part of the regulatory dossiers [47, 58, 59].

Theranostic cargos need bioanalytical and imaging-specific assays. Fluorescence stability and photobleaching assays are used to investigate optical probes. MRI Relaxometry gives quantitative readouts of contrast agent, and R1/R2 mapping gives a potential for in vitro and in vivo quantification of uptake for iron oxide or gadolinium-based tracers. Radiolabeling for PET/SPECT requires strict requirements for radiochemical purity and radiochemical yield, as well as records of stability and purification. Cross-validation of imaging signals with chemical quantification methods gives one more confidence in biodistribution data [60].

Good analytical practice for novasomes includes the use of orthogonal assays with good acceptance criteria. A significant limitation in the current literature is the lack of standardisation of data reporting; many studies report particle size and encapsulation efficiency, but do not report other important parameters such as lamellarity, bilayer rigidity, long-term stability or inter-batch variability, and even then, the different analytical protocols, instrument settings and sample preparation techniques can lead to significant differences in the performance characteristics in different studies of similar formulations. Harmonized analytical standards and clearer regulatory guidance for vesicular nanocarrier systems are needed. Routine reporting should contain information about the make and model of the instrument, measurement setting, preparation of the sample, number of replicate measurements, and processing of data. Inclusion of representative raw data files or supplementary spreadsheets helps to provide reproducibility and expedite regulatory review. Specific analytical methods measured parameters, purposes, and suggested target values for formulation screening and release testing are summarized in the following Table 3.

**Table 3:** Analytical techniques used for physicochemical characterization, quality assessment, and release testing of novasome formulations with recommended acceptance criteria

Analytical method	Measured parameter	Purpose	Target value	Sample prep notes	Reference
DLS (photon correlation)	Mean hydrodynamic diameter, PDI	Size control, batch uniformity	Diameter 80–250 nm; PDI ≤ 0.2 for monodisperse; ≤0.3 acceptable	Dilute in filtered buffer; 0.1-1 mg/mL; avoid dust; report temp/angle	[45]
Zeta potential (electrophoretic)	(mV)	Colloidal stability indicator	≥	± 25	[46, 47]
Cryo-TEM/TEM	Morphology, lamellarity	Visual confirmation of structure	Representative micrographs showing expected lamellarity; scale bar included	Vitrify on lacey grid; low-dose imaging; include multiple fields	[48, 49]
DSC	Tm, ΔH	Bilayer order and payload interaction	Consistent Tm shifts vs. blank; report onset, peak, endset	Use sealed pans; scan rates 5–10 °C/min	[50]
SAXS/WAXS	Bilayer spacing, thickness	Lamellarity and bilayer structure	Repeat spacing and bilayer thickness are consistent with the design	Use concentrated samples; match buffer scattering	[61, 62]
FTIR	Functional group shifts	Drug-lipid interaction confirmation	Peak shifts consistent with known interactions	Dry film or ATR mode; baseline corrected	[51]
HPLC	Drug content, impurities	Quantification and assay validation	Assay accuracy 98–102%; precision RSD ≤ 2%	Protein precipitation or solvent extraction; validate recovery	[54]
LC-MS/MS	Trace quantification, metabolite ID	Bioanalysis and complex matrices	LLOQ suitable for PK needs; matrix effect < 15%	Spiked calibration and QC; internal standard needed	[55]
Franz diffusion/IVRT	Cumulative permeation (%)	Topical release and permeability	Sink conditions; linearity for IVRT systems; report flux ± SD	Use validated membranes or human/ex vivo skin; 32 °C for skin	[56]
Release kinetics modelling	Fitted model parameters	Mechanistic interpretation	Goodness-of-fit R² ≥0.95 for chosen model; report n and k	Use cumulative data and log-transform where applicable	[63]
Stability testing (ICH)	Size, EE, potency, residual solvent	Shelf-life and storage conditions	Compliance with ICH zones; no significant change over time	Use ICH storage chambers; include photostability cells	[58, 59]
MRI relaxometry/ Radiochemistry	R1/R2, radiochemical purity (%)	Imaging payload quantitation	Radiochemical purity > 95%; relaxivity consistent with tracer specs	Calibrate with standards; perform purification checks	[60]

## Targeting strategies and skin/tumor penetration

Targeting strategies for novasomes are concerned with local better deposition in skin as well as with better tumor selectivity following intralesional delivery. Passive mechanisms take advantage of particle physics and the anatomy of the skin to favour their accumulation at or close to the lesion. Active strategies make use of surface ligands for receptor binding to tumor or stromal cells. Stimuli-responsive elements introduce a temporal control dimension, which releases payloads based on tumor-specific biochemical cues. Adjunctive physical and chemical enhancers have more ways to gain tissue access and residence. The use of these tactics integrated provides support for localized efficacy, but limits the amount of systemic exposure [8, 11, 64].

Passive targeting is highly dependent on particle size and deformability. Particles with a size fraction less than ~ 300 nm exhibit improved follicular accessibility and deeper skin appendage deposition compared to larger-sized particles [64, 65]. Follicular reservoirs are drug depots and allow a sustained-release response to adjacent epidermal and dermal cells. Deformable vesicles containing edge activators or ethanol exhibit greater ability to penetrate the stratum corneum through temporary shape-change and squeeze through intercellular spaces. These vesicular characteristics increase local retention of the agent at the lesion site and extend the contact time of the therapy. Size control and mechanical softness are, therefore, powerful formulation levers for passive targeting of cutaneous tumors [65].

Active targeting helps to add molecular selectivity to the passive accumulation. Common ligands include short peptides, monoclonal antibodies or antibody fragments, small molecule ligands such as folate and nucleic acid aptamers [66]. Peptides offer short targeting moieties, low immunogenicity, and easy synthetic alteration [67]. Aptamers provide high affinity as well as chemical stability with flexible conjugation chemistry [68]. Antibody-based ligands provide high specificity and high avidity against overexpressed tumor markers at the expense of larger size and, in some cases, high immunoreactivity. Conjugation strategies for novasomes usually employ PEG spacers, maleimide-thiol coupling, carbodiimide (EDC/NHS) chemistry, or click chemistry modalities for placing ligands at controlled distances away from the surface of a vesicle. The avidity and uptake of antigen by cells are controlled by ligand density tuning. Low density of ligand may underperform in terms of receptor engagement, and excessive ligand density may lead to steric hindrance and/or rapid clearance. Optimal presentation of ligands achieves a balance of multivalency, steric accessibility, and colloidal stability for maximal target engagement in cutaneous tumor microenvironments [66–68].

Stimuli-responsive release systems give spatial and temporal control over the payload release. The mildly acidic environment of the tumor and higher levels of protease activity in skin tumors can be triggered to use pH-sensitive lipids and enzyme-cleavable linkers [69, 70]. pH-responsive lipids that destabilize the bilayers in acidic environments allow for the release of the cargo in acidic tumor interstices. Protease-sensitive peptide linkers are broken down in the presence of matrix metalloproteinases or other tumor-associated proteases, to dissociate surface shields or to open the shells of nanocarriers. Thermo-responsive polymers or lipids are applied to respond to localized hyperthermia or externally applied heat to phenomena such as phase change and release. These chemistries allow for increased payload concentration at the lesion, and decreased exposure of the non-targeting tissues for improved therapeutic index for cytotoxics and radiosensitizers [69–71].

Barrier modulation and physical enhancement strategies can be combined with optimized vesicle design to further improve skin penetration. Chemical permeation enhancers such as terpenes, oleic acid, and short-chain alcohols temporarily increase stratum corneum lipid fluidity and improve the partitioning of lipophilic cargos into the skin layers [72]. Microneedles induce micropores via holes or place payloads onto the epidermis and superficial skin dermis, and the nanosystem can be used to bypass the stratum corneum completely and almost instantaneously [73]. Iontophoresis involves the application of low-level electric current, which drives the charged molecules and particles across the layers of skin, and can be coupled with vesicular systems to aggressively enhance the local uptake without mechanical disruption [74]. Combining physical pretreatment approaches with optimized novasome formulations can increase drug deposition at tumor sites while minimizing invasiveness. Safety and tolerability issues determine which enhancers are chosen and the dosing regimens that are used in the clinic [72–74].

Design trade-offs determine the practical implementation. Increased deformability may increase the penetration but may decrease the storage stability. High ligand density enhances the binding of targets but may result in increased rapid recognition of the target by the immune system or aggregate formation. Stimuli-responsive linkers increase the release precision, synthetic complexity, and regulatory scrutiny. The manufacturing required for intradermal delivery with microneedle-assisted delivery must be aseptic. Iontophoretic adjuncts require standardized dosing of current and electrode design to run in a reproducible manner. These operational constraints will recommend an incremental development pathway. In early-stage studies, physicochemical robustness, reproducible manufacturing, and quantitative measures of follicular and tissue distribution should be the main priorities. More advanced work can proceed to device integration and small human feasibility studies using imaging-guided readouts [11, 64, 66, 74].

**Fig. 3:**
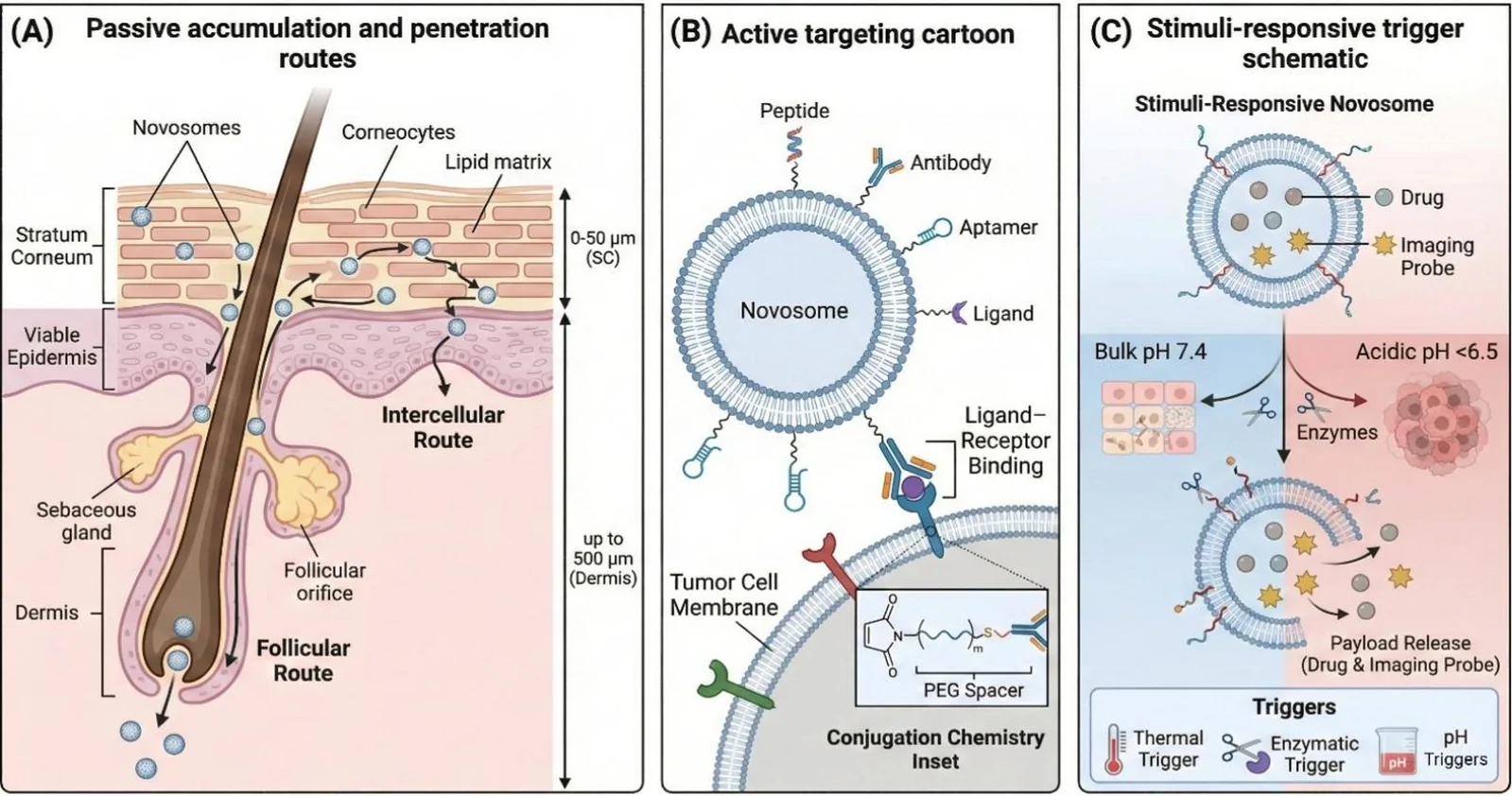
Passive and active targeting strategies of novasomes, illustrating follicular deposition, receptor-mediated uptake, stimulus-responsive release, and enhanced penetration in cutaneous oncology

In conclusion, effective targeting of cutaneous tumors with novasomes is an approach that combines the passive size and deformability tuning, active ligand-mediated targeting, stimuli-responsive release mechanisms, and adjuvant penetration technologies. Figure 3 shows passive and active targeting mechanisms of novasomes. Rational selection between these modalities provides the potential to have a customized therapeutic profile for topical or intralesional treatment formats. Consistent reporting of the particle size, ligand density, trigger sensitivity, and local tissue concentration will boost the cross-study comparison and clinical translation of novasome-based cutaneous oncology applications.

## Diagnostic and theranostic integrations

The incorporation of diagnostic moieties into novasome platforms enables real-time monitoring of drug distribution and treatment response. Theranostic novasomes combine therapeutic payloads and imaging probes in a single construct to enable early detection, image-guided dosing, and response-guided therapy. By this integration it is possible to minimize dose uncertainty and to speed up clinical decision making in the field of localized cutaneous oncology. Combining local deposition, retention, and functional response information enhances the likelihood of on-target efficacy and reduces systemic exposure to cytotoxic agents [9, 75, 76].

The imaging payloads are based on lasers, providing high sensitivity to have convenient intraoperative readouts. Near-infrared fluorescent dyes, such as indocyanine green (ICG), and tumor-targeted fluorophores enable visualization of superficial lesions and surgical margin mapping. The strategies to be employed in loading include direct bilayer inclusion of lipophilic dyes and aqueous core entrapment of hydrophilic labels. The stability of optical probes is influenced by both the carrier microenvironment and the physicochemical properties of the dye. Lipid-rich bilayers undergo encapsulation, which usually minimizes photobleaching and local retention. Real-time fluorescence guidance for excision and local dosing checks and controls in intralesional procedures has been made possible by using bright, photostable fluorophores and appropriate optical filters [9, 75].

Magnetic resonance contrast agents provide high-resolution anatomical and functional imaging. Complementary T1 and T2-contrast is produced using gadolinium chelates and Iron-oxide nanoparticles. Superparamagnetic iron oxide nanoparticles (SPIONs) enable the simultaneous integration of MRI contrast enhancement and drug delivery. The SPION cores may be coated or enclosed by Novasome bilayers in order to be observed by MRI and to release therapeutics. The gadolinium chelates may be either encapsulated or conjugated to the wall of the vesicle to enhance their relaxivity in the lesion site. The clinical translation of the materials should be granted a safety and clearance profile, decreasing free metal exposure, and increasing the in vivo stability through the application of chelation or encapsulation approaches [9, 77, 78].

Nuclear Imaging Payloads Sensitivity and quantification of the in-depth tissues and entire body biodistribution studies. PET and SPECT radionuclides have quantitative readouts that are applicable in biodistribution images of translation and dosimetry. Radiolabelling of novasomes can be based on chelator-based radiosource (i.e., radiometal) 64Cu or 89Zr or surface-based labelling of technetium isotopes or iodine isotopes. Radiochemical stability in the physiological conditions and radiochemical purity are obligatory reporting items. Dual-labelling techniques are cross-modal validation, which involves sensitive positron emission tomography (PET) quantification with high-resolution magnetic resonance imaging (MRI) or optical overlays to guide surgery or plan dose [79–81].

Physicochemical complementarity is the basis of loading strategies of imaging probes. Lipophilic agents separate into the lipid bilayer on lipid dissolution, and the radiotracers and chelates of the preparation are confined in the aqueous core on hydration or post-formation loading with ion gradients. The ability to maintain accessibility of the probe and eliminate quenching by proximity to the bilayer is made possible through surface conjugation-peg spacers or bifunctional chelators. Imaging signal cross-validation with chemical quantification gives the biodistribution claims more strength and is of regulatory importance to enable IND enabling studies [75, 78, 80].

Although imaging-enabled novasomes have many benefits, there are several technical limitations that need to be taken into account. The incorporation of fluorescent dyes, magnetic contrast agents or radiotracers in surfactant-enriched bilayers might affect the intensity of the signals and the sensitivity of the detection [82]. Local high concentrations of fluorescent probes can cause self-quenching effects and entrapment of probes in packed vesicular membranes can slow the mobility of the probes and thus help to attenuate the signal [83]. These effects can constrain the accuracy for real time imaging, especially for small lesions and low abundance targets. Likewise, the signal that is detectable may not be directly proportional to the concentration of payload due to probe leakage, degradation and microenvironmental effects. Hence, loading of probes, stability of signals, and cross validation with other complementary analytical methods are still crucial to ensure reliable clinical monitoring and translational development [82–84].

Novoasome systems are further able to perform active molecular read-out by means of biosensing as well as biomarker-capture capabilities. Novasomes are functionalized in a way on their surface by the usage of antibodies, peptides, or aptamers to create capture platforms that selectively bind specific cancer-specific proteins, including those of extracellular vesicles on the tumor surface. Aptamer Enabled Sensing Affinity sequence control, and a small footprint of dense surface functionalization. Local signal amplification can be achieved using immobilized reporter dyes/activable probes and results in a localized response on biomarker engagement. These nanoscale sensors enable non-invasive sampling of the tumor microenvironment, and repeat dosages may be changed with respect to molecular response [81, 85, 86].

Theranostic novasomes-Enabling viable clinical applications-cases of cutaneous oncology. Image-guided intralesional therapy entails the application of real-time images in order to ensure that the deposition is accurate and to measure the local retention over time. Fluorescence-guided surgical margin mapping can help facilitate more complete surgical resection in a small volume of healthy tissue and with real-time capability of local dose into residual margins. The co-registered imaging of PET/MRI is an aspect that aids in translating preclinical research to clinical research by providing information on the dose, leakiness, and off-target retention of the target site in the tissues. These readouts enable the use of adaptive dose escalation and sentinel dosing mechanisms that put patient safety first and enhance the signal detection of therapy in the earlier trials [75, 76, 80].

**Fig. 4:**
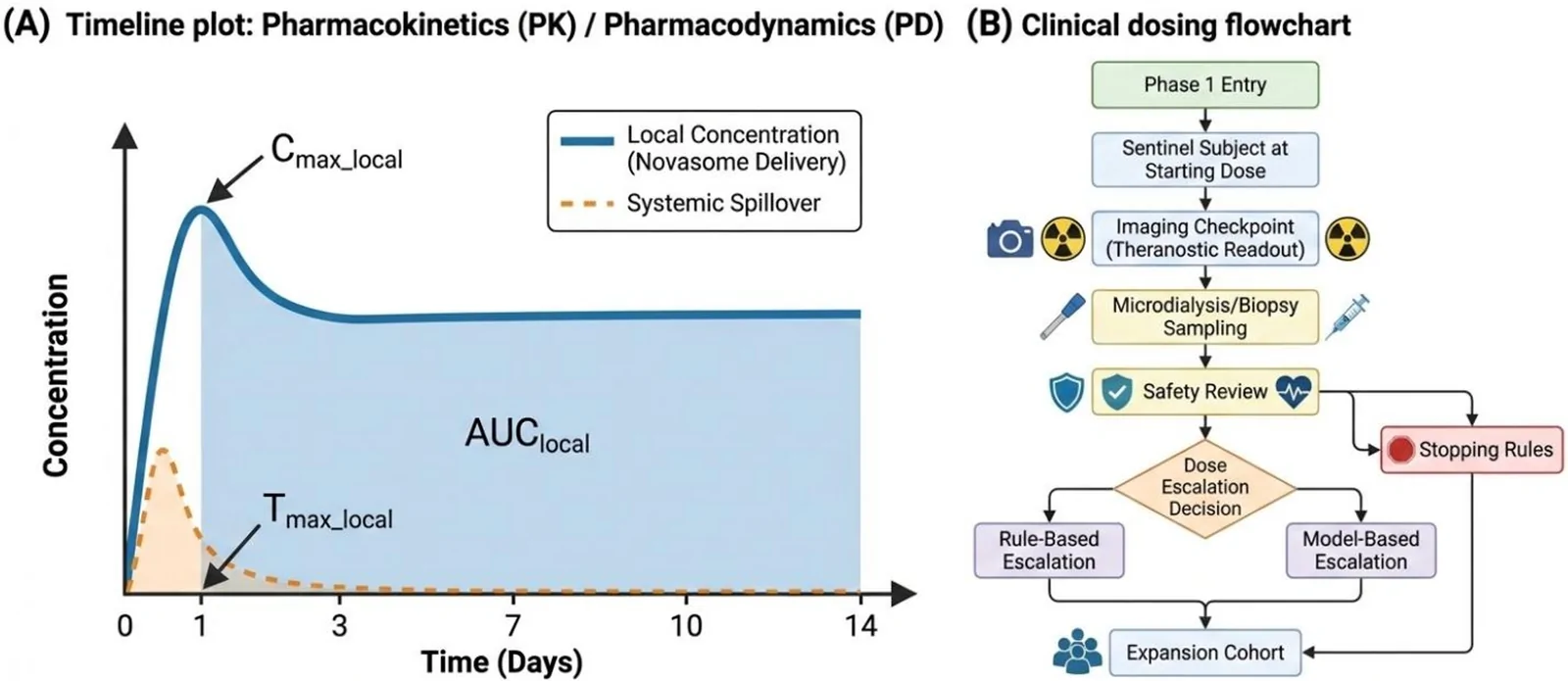
Theranostic novasome platform illustrating co-delivery of therapeutic and imaging payloads for biodistribution monitoring and PK/PD-guided dosing in cutaneous oncology

The important theranostic design players are stability, safety, and regulation criteria. Imaging probes are required to be stable over time in terms of their storage and their location within the biological environment. Pharmacology data reports would be required to have radiochemical stability data as well as relaxometric stability data. Other layers of control include contrast agents and radiolabels, which require toxicology and biodistribution studies that are specifically required. Label-free/biodegradable imaging agents can be used to facilitate regulatory requirements, and full analytical characterization can be used to speed up translational review routes [77, 78, 80]. Early cross-modal validation should be incorporated into the developmental pathway to strengthen translational confidence. A pragmatic translational strategy should incorporate rigorous physicochemical characterization, orthogonal imaging validation, in vivo biodistribution studies, local efficacy assessment, and safety evaluation. The increased reproducibility and clinical adoption of novasome-based theranostics in cutaneous oncology will be boosted through open reporting of probe chemistry, labelling methods/signal-dose relationships. Figure 4 shows the strategy of theranostic novasomes that co-encapsulate therapeutic and imaging agents as well as representative multimodal imaging readouts.

While theranostic novasomes are an appealing strategy for combining diagnosis and therapy, a number of issues must still be addressed: Co-encapsulation of imaging agents with therapeutic payloads can impact the stability of the vesicles, the release of the therapeutic agents, and the signal intensity, which in turn can reduce imaging sensitivity. In addition, multimodal theranostic systems are more complicated to analyze, evaluate, and review for toxicology and regulatory requirements than conventional drug delivery systems. Future development should aim to balance the diagnostic function with the simplicity of formulation, safety and manufacturability.

## Dosing strategies, pharmacokinetics, and PD considerations

Localized dosing routes are significant in the therapeutic design of cutaneous oncology. Topical administration in forms of creams, gels, or patches provides a noninvasive route of administration with the best situation of drug contact with the epidermis and superficial dermis [87, 88]. Topical formulations are suitable for small lipophilic drugs and formulations that would promote stratum corneum permeation. Dosing frequency for topical forms is dependent upon residence time in the skin, release kinetics, and tolerability to the skin. Once-daily to twice-daily regimens are common for sustained contact formulations. Semi-solid carriers with controlled release allow for the reduction of the frequency of application and hence improve patient adherence [88, 89].

Intralesional injection places the drug directly into the tumor or peritumoral tissue. This route yields high local concentrations and minimal systemic exposure when deposition is precise [90]. Volume, concentration, and injection technique determine the drug’s distribution within the lesion. Repeated intralesional dosing is useful for maintenance of control of accessible lesions, as well as that of immunomodulatory agents with a local action. Sentinel dosing strategy and careful safety monitoring are the foundation for early clinical testing for intralesional therapeutics [91].

Microneedle-assisted administration is a link between topical administration and parenteral administration. Microneedles create microchannels and/or deposit a payload into the epidermis and superficial dermis with minimal pain [87, 92]. Dissolvable or coated microneedles offer delivery of nanoparticles, proteins, or nucleic acids without the large-bore injections. Dosing regimens for microneedle systems are aligned with target residence time and depot dissolution rates. Microneedle patches permit the repeated home administration for chronic local therapy, giving assured sterility and device stability [87, 93].

Release kinetics tuning is an important dosing strategy. A burst release results in high local drug exposure as soon as after application or injection. Burst profiles are appropriate for short-acting cytotoxic and ablative interventions. Sustained release increases the exposure duration of the therapeutic over a time frame of days to weeks from a single application and reduces the dosing frequency. Pulsatile release provides defined boluses at determined intervals and periodically corresponding to some of the therapeutic windows that require peak exposures separated by recovery periods. Matching the release profile to the therapeutic window is important to improving efficacy and reducing both local and systemic toxicity [94, 95].

Formulation controls enable specific kinetic profiles. Bilayer Rigidity, Encapsulation Depth, and Polymeric Matrices Determine Exit Rate of Novasomes Formulations. Surface modifications and stimuli-responsive linkers are available for release by triggering on exposure to pH, enzymes, or thermal stimuli in the tumor microenvironment. The design choices should align with the pharmacology. Fast-release carriers favor agents that have short target engagement requirements. Drugs having long intracellular action are suitable for long-release depots [94, 96].

The PK/PD modelling for localized therapy breaks away from paradigms that apply to systemic therapy. Local concentration is the most important factor of the acting effect, where the effect is expressed at the site of the action. Systemic exposure enables the context of risk, informing off-target risk. Models should include a compartment for stratum corneum, viable epidermis, dermis, and tumor interstitium to represent local gradients well [96, 97]. Residence time at the target site, as well as the rate of elimination to systemic circulation, are important parameters. Modeling should be done for binding to tissue components and local metabolism in the skin (enzymes of the skin).

Sampling strategies need to have the ability to provide site-specific read-outs (PK and PD). Dermal microdialysis offers a way to sample free drug in the interstitial fluid continuously and for direct determination of the local pharmacokinetics [96, 98, 99].

Correlating in vitro release and ex vivo permeation of drug products with in vivo site exposure remains, in practice, considered a practical priority. Franz diffusion and IVRT data are available to provide screening-level data on release and permeability for topical formulations [100, 101]. For local exposure, quantitative IVIV correlations will be helpful to improve predictability. Differences in the sink conditions and the use of standardized skin models with validated analytical methods will help to improve the comparability of different experiments, as well as reduce animal and early human exposure risk [100, 102].

First-in-human cutaneous applied studies have a dose escalation design, which should focus on a primary safety calculation while enabling an informative pharmacology to be performed. Sentinel dosing of the 1st subject/cohort appears to give early signals of the safety and reduce the risk for exposure in the early stage [91]. Dose escalation schemas usually use rule-based or model-based approaches. Model-based Bayesian designs are more efficient in the sense that it.

fixate their estimate of dose-toxicity to information that may have previously been learned and continually update their estimate. Safety endpoints for safety tolerance and safety signals are the local tolerance (erythema, ulceration, necrosis) and systemic adverse events and safety signals for laboratory markers of immunogenicity for the engineered carriers [91].

Loading dose using imaging is an aid to the adaptive escalation and a means of dose interpretation. Theranostic labels used in novasomes have the possibility of being used to directly measure deposition and leakage at the local level, as well as the systemic circulation. Imaging checkpoints can be used to define the escalation boundaries by demonstrating an adequate retention locally at candidate doses and the absence of excessive spillage through the rest of the body. Correlating surrogate local concentration data collected in the imaging studies with data collected from microdialysis or biopsy helps to improve PK/PD inference and assists in dose selection for future trials [90, 103].

Data collection practices and modelling practices of translational programs need to have hard standards. Reported metrics will need to include local peak concentration (C_max_ local), area under the concentration-time curve at the lesion site (AUC local), systemic exposure (AUC systemic), residence time, and local PD. Statistical analysis plans should predefine PK/PD endpoints as well as stopping safety rules. Early engagement with regulatory authorities helps in ensuring that study design is in line with expectations for combined-device or drug-device products and also for imaging agent co-development [91, 96].

In summary, novasome-mediated cutaneous therapy dosing strategies incorporate route, release feature, and site-of-action PK/PD understanding. Topical, intralesional, and microneedle-assisted each has its unique advantage. Matching the release to the therapeutic windows and enriching sample modalities, i.e., microdialysis, imaging, increases confidence in translation. Exemplary dose-escalation schemes using sentinel dosing and radiologic surveillance sites minimize risk and enable more timely discovery of local regimens of efficacy.

## Preclinical and clinical evidence

Preclinical evaluation of novasomes formulations is related to a hierarchy of models that are designed to build evidence of skin delivery of local efficacy and safety. In vitro assays are initiated using 2D cancer cell lines, which are representative of cutaneous malignancies. Common choices include lines of models of malignant melanoma, squamous cell carcinoma, and basal cell carcinoma. These monolayers provide high-throughput cytotoxicity screening as well as mechanism of action readouts using viability, apoptosis, and clonogenic assays [7, 104]. Uptake studies on cells (using confocal microscopy) are useful in determining internalization kinetics and subcellular localization of therapeutic and imaging cargos [8, 104]. Migration and invasion assays help put in functional context the anti-metastatic potential, and also the stromal interaction. Controlled in vitro screening enables candidates for cargo and surface chemistry prioritization for further testing [7, 8, 104, 105].

The three-dimensional models provide a better approximation to tumor architecture and diffusion barriers. Spheroids recreate the power of gradients of oxygen, nutrients, and drug penetrations that are absent in monolayers. Skin equivalent models and epidermis reconstruction in the contribution of stratum corneum barrier properties in penetration and retention studies. These platforms allowed the cytotoxicity and the penetration and local biomarker changes after the topical or microneedle-assisted application of novasomes to occur in parallel [4, 5]. Use of human-derived keratinocytes or patient-derived tumor tissue for ex vivo explant cultures provides more clinical relevance regarding translation decisions [106, 107].

Ex vivo penetration and release studies from human skin and Franz diffusion studies give data on topical concentration, penetration, and release. Full-thickness human skin or Dermatome human skin samples are made available when used to measure drug deposition in epidermis and dermis, in controlled sink conditions. These studies provide information on flux and lag time as well as cumulative deposition parameters formulation adjustment to target site exposure [8, 108]. Ex vivo histology following exposure is a source of morphological safety information and also helps to complement biochemical endpoints. The combination of Franz cell results, imaging, and chemical quantification is better used to predict retention in vivo novasomes [8].

Therapeutic efficacy, biodistribution, and tolerability are tested in vivo using models of tumors. Common in vivo paradigms include subcutaneous xenograft models, orthotopic skin tumor grafts, and chemical-induced carcinogenesis models. Subcutaneous models give the chance of standardized endpoints in terms of tumor volume, as well as the possibility of imaging follow-up. Orthotopic skin models are a better representation of the tumor microenvironment and the local immune context. Chemically induced mouse models offer the possibility to recapitulate the process called multistage carcinogenesis, and can be used as long-term endpoints for prevention and progression studies [109, 110]. Endpoints in these studies are tumor volume reduction, time-to-progression, survival, histopathology, and imaging-based response measures using optical, MRI, or radionuclide readouts for theranostic cargos [105, 107].

Although subcutaneous xenograft models are widely used because of their simplicity and reproducibility, they do not fully replicate the complex architecture of human skin, including the stratum corneum barrier, follicular pathways, vascular organization, and local immune microenvironment. Therefore, positive results in terms of drug penetration and therapeutic responses in xenograft models do not necessarily reflect clinical performance with topical or intralesional administration. By contrast, ex vivo human skin models are more clinically relevant and can give information about permeation, retention and barrier interaction but do not possess systemic and immunological elements. Orthotopic and patient-derived tumor models provide better biological relevance but are more time consuming and complex to work with. Thus, current xenograft studies should be interpreted with caution and future studies to translate from bench to clinic should be carried out using ex vivo human skin studies as well as advanced in vivo models to better predict clinical outcome in cutaneous oncology [111].

Translational Potential Examples of Novasomes: Preclinical Curcumin-containing novasomes were demonstrated to have a controlled release, enhanced skin deposition, and tumor growth inhibition in Ehrlich carcinoma models after topical or peritumoral injection [105]. Quercetin-Loaded novasomes with selenium modification gave superior cellular uptake, apoptotic signaling, and improved antitumor efficacy in rodent models [104]. Formulations of fluconazole and terconazole novasomal were demonstrated to have an improved deposition on the skin, microbiological results, and (in one exploratory clinical test) clinical cure of napkin candidiasis in infants [7, 8]. Some recent drug repurposing and anticancer investigations are nintedanib- and other small molecule-loaded novasomes with superior bioavailability and antitumor activity in preclinical and initial translational reports [106, 107]. These studies always find the high encapsulation efficiency and increased local retention in comparison to the conventional formulations [7, 8, 104, 105, 107].

Immunological readouts are also increasingly being designed during preclinical pipelines for Novasome therapeutics. Tumor microenvironment modulation is an efficiency-determining factor for many anticancer agents. Studies follow the patterns of immune cell infiltration, including CD8 + T cells, regulatory T cells, tumor-associated macrophage phenotypes (M1/M2), Dendritic cell activation, and Natural Killer cells. Cytokines and chemokines (e.g. IFN-g, TNF-alpha, IL-6, IL-10, CCL2) functional readouts of immunological activation or restraint after local novasome treatment [112, 113]. Nanocarriers can be engineered to carry immunomodulatory cargos or combine delivery with adjuvants in order to re-exert local immunity. A combination of immunological endpoints with standard measures of tumor volume provides more mechanistic information and will inform strategies for combination with checkpoint inhibitors and other immunotherapies [112, 113].

Safety and tolerability data are from local histopathology studies, systemic toxicology, and biodistribution studies. Local endpoints that include erythema scoring, epidermal/dermal integrity, inflammatory cell infiltrates, and necrosis. Systemic endpoints include: blood chemistry, Hematology and histology of off-target organs. Biodistribution studies with radiolabels, fluorescence, or MRI contrast agents report spillage into the systemic circulation and accumulation in the organs for clearance profiling. On the other hand, these data are the basis of the IND enabling packages and clinical trial safety monitoring plans [106, 107, 113].

Clinical evidence for novasomes platforms is still concentrated in the field of dermatology and topical anti-infectious, with some exploration studies on humans toward the application of novasomal gels with antifungal properties. A novel formulation of terconazole, novasomal randomized clinical exploration has shown complete clinical and mycological cure in a small group of infants with napkin candidiasis and good local tolerability [7]. Beyond the topical antifungals, the clinical development in the oncology field remains in its early stages. Investigator-led early phase projects and recent translational publications are indicative that there is a growing interest in novasomes for topical anticancer and adjuvant vaccine use [106, 107]. However, industrial patents and proprietary portfolios are evidence of more general commercial activity. Notable patent filings are made that describe novasomes vesicles and uses thereof for topical delivery and cosmetics, and vaccine adjuvants, also US patent applications and granted patents define composition and manufacturing claims [108, 110]. These patents support the translation in the industrial setting and present composition of matter and process claims that need to be considered in the development plan [108, 110].

Collectively, preclinical studies have demonstrated that novasomes enhance drug loading, improve local retention, and enable the co-delivery of therapeutic and diagnostic payloads. A broad focus on robust and standardized preclinical endpoints, immunological profiling, and orthogonal imaging validation will ease clinical translation. sRepresentative preclinical and clinical studies evaluating novasome-based systems and their translational relevance are summarized in Table 4.

**Table 4:** Representative preclinical and clinical studies on novasome-based systems

Novasome-based system / payload	Study type / model	Key outcome reported in the manuscript	Translational relevance	Ref.
Terconazole-loaded novasomes	In vitro characterization, in vivo assessment, and exploratory clinical experimentation	Improved topical delivery of terconazole; exploratory human use was reported	Clinical/exploratory human evidence for novasome feasibility in topical delivery	[7]
Fluconazole-loaded novasomes	Preclinical topical delivery study	Enhanced topical delivery of fluconazole for fungal infections	Supports topical performance of novasomes; no clinical oncology data	[8]
Selenium-plated novasomes loaded with quercetin	In vitro optimization and in vivo radiobiodistribution in Ehrlich tumor-bearing mice	Fine-tuned pharmacokinetics and tumor uptake of quercetin	Preclinical oncology evidence	[104]
Curcumin-loaded pro-novasomes	In vitro characterization, statistical optimization, and subcutaneous Ehrlich carcinoma mouse model	Enhanced skin deposition and tumor growth inhibition	Preclinical skin cancer evidence	[105]
Fluvoxamine-loaded novasomes	Pharmacokinetic evaluation	Demonstrated novasome applicability for systemic delivery in a non-oncology setting	Preclinical/translational novasome evidence, not oncology	[106]
Nintedanib-loaded novasomes	Bioavailability and therapeutic efficacy evaluation in non-small cell lung cancer	Suggested improved bioavailability and therapeutic efficacy	Oncology-focused translational evidence, but not cutaneous	[107]

## Challenges, future perspectives, and translational roadmap

The successful clinical translation of novasome platforms requires coordinated efforts to address scientific, technical, and regulatory challenges. Stability under storage and physiological conditions is a major challenge from a scientific point of view. Novasome bilayers are sensitive to oxidation, hydrolysis, and desorption of excipients. Preservation of encapsulation efficiency and particle size over the shelf life is of major importance for predictable dosing as well as regulatory acceptability [114, 115]. Scale-up and technology transfer remain significant barriers to clinical translation. Lab methods employing solvent evaporation and batch sonication do not correlate at all with industrial continuous manufacture. Reproducible particle size, PDI, and drug loading from batch to batch is one of the challenges that requires strong process controls and validated scale-out strategies [114, 116].

Reproducible targeting and functional performance require demanding characterization and standardized reporting. Minor variations in the surfactant chemistry, PEG chain length, or ligand density can change the biodistribution and immunogenicity. A lack of harmonization of minimum reporting is at risk of making the results non-reproducible between laboratories and delaying the decision in translation [117, 118]. Regulatory ambiguity is a development risk added on. Current regulatory approaches apply a case-by-case approach of safety evaluation of nanomaterials, and may require extensive orthogonal data sets for defining safety and comparability. Early regulatory involvement and the use of reference characterization cascades to minimize the risk of uncertainty and speed up the review [115, 119, 120].

Standardized experimental reporting and comprehensive analytical characterization form the foundation for future progress. Adoption of minimum reporting checklists, including full composition, solvents, and excipient grades, exact methods, particle size/PDI distributions, zeta potential, lamellarity, encapsulation efficiency (EE%), drug stability, in vitro release conditions, and in vitro model details, will allow for comparison between studies [114, 117]. Animal model descriptions should include such information as strain, sex, tumor model, implantation site, tumor size upon treatment, dosing schedule, and primary endpoints. Supplying Raw Data Files and SOP Links in Supplementary Materials, To Promote Reuse and Meta-analysis [117, 121].

Research questions are prioritized to focus on translationally impactful research gaps. Quantitative biodistribution in human skin and lesion microenvironments has the highest priority. Micro dialysis, the imaging-based dosimetry, and biopsy analyses can be used to define the local C_max_ and AUC at the site of action and clear the systemic spillover [122]. Imaging-guided dosing trials based on theranostic labels will be able to give direct evidence of target site retention and rational dose selection. Comparative studies between in vitro release and ex vivo permeation and correlation with in vivo exposure to the lesion will increase IVIV correlations for topical and intralesional routes [120, 122].

Establishing multicentre preclinical consortia could improve reproducibility and accelerate regulatory readiness. Reference labs such as the US NCI Nanotechnology Characterization Laboratory (NCL) and the European Nanomedicine Characterization Laboratory (EUNCL) and their validated assay cascades and SOPs, are in line with expectations of regulators [117, 120, 122]. A coordinated consortium model can be utilized to pool the expertise for standardized assay performance, interlaboratory ring trial, and centralized SOP curation. These shared resources will reduce the duplication and increase the confidence of the preclinical packages submitted to the regulators [116, 123].

Standard operating procedures are needed in several interfaces. SOPs covering material sourcing, particle preparation, sterility assurance, endotoxin testing, residual solvent analysis, and analytical assays will facilitate regulatory submissions. Accessible SOP templates and annotated method checklists are publicly accessible and speed up the adoption of SOP and improve the traceability. Accession numbering of SOP for manuscripts and submissions to the regulatory authorities will make audit trails and inspection readiness easier [117, 121].

Combination therapy opportunities are a high-value translational frontier. Novasomes are well-suited to be used for the co-delivery of immunomodulatory agents, photosensitizers for photodynamic therapy (PDT), and small molecules with a synergistic action with immune checkpoint blockade. Nanocarrier-based co-delivery has the potential to localize adjuvants and antigens to the lesion and minimize toxicity to the rest of the body. Preclinical data are in support of combinations of nanoparticle carriers with PDT and checkpoint inhibitors to enhance immunogenic cell death as well as remodel the tumor microenvironment for better systemic responses [118, 124]. Such combination strategies call for integrated PK/PD and immunological endpoints in earlier studies [124].

A translational roadmap should clearly define both short-term and long-term milestones. Short-term goals include: adoption of standard reporting checklists, ring trial validation of important assays, and generation of strong IVIV correlations for lead formulations. Medium-term goals include feasibility studies in humans commensurate with safety, imaging-guided dosimetry and sentinel dosing schemas, and first-in-human studies. Longer term goals include investigator-led combination trials with Novasome delivery in combination with immunotherapies, PDT, or localized radiation, as well as developing algorithms for precision dosing based on imaging and microdialysis readouts [116, 120, 124].

Both scientific and non-scientific factors contribute to commercial and clinical barriers. Manufacturing cost and cost of goods may be a limiting factor to commercial viability for complex dual-payload systems. Intellectual property landscapes of carrier chemistries and imaging agents may constrain freedom to operate and partnership models. Clinical adoption requires demonstration of definite benefit over the extant standard of care, and manageable logistics for dosing, imaging, and device co-administration. Mitigation strategies include early value proposition analyses, working with imaging companies, and incremental product development beginning with simpler and lower-barrier topical applications [114, 115, 125].

Data sharing and data repositories are the keys to community learning as well as redundant experiments. Public deposition of standardized datasets, of negative results, and full SOPs in repositories such as Zenodo, Figshare, and domain-specific databases promotes transparency and allows for meta-analysis. Dedicated nanomedicine repositories and databases aligned using ontologies such as eNanoMapper and the EU Observatory for Nanomaterials facilitate a measure of structure to the data produced on hazard, exposure, and performance. A community mandate for negative-result deposition will result in less publication bias and a more rational design in a shorter time [121, 126].

In closing, coordinated standardization, consortia, imaging-enabled characterization, dosing strategies, and open data practices will expedite novasome translation. Strategic investment in assay harmonization and early interaction with regulators will reduce translational friction. The prioritization of clinically relevant endpoints and combination strategies will place novasomes in a position to have an impact in cutaneous oncology and beyond.

### Toxicity, immunogenicity, long-term safety, and regulatory challenges

Despite their promising therapeutic potential, multifunctional novasomes present several safety and regulatory challenges. Targeting ligands, imaging probes, and stimulus-responsive components can increase formulation complexity and influence the overall toxicological profile. Non-ionic surfactants are typically regarded as biocompatible, but chronic exposure can cause inflammation, irritation or changes in the compatibility of tissues. Surface modified systems can also interact with immune components with potential impact on long term tolerability. Moreover, knowledge of long-term accumulation, biodistribution and chronic exposure of multifunctional payloads is scarce. Regulatory-wise, theranostic formulations need to be extensively characterized, reproducibly manufactured, analytically validated, and be subjected to thorough safety evaluation. These problems will need to be solved to enable the successful clinical translation of novasome based precision oncology systems [127].

## Conclusion

Novasomes platforms for cutaneous oncology treatment, a surfactant-rich multilamellar vesicles represent a platform that is located at the crossroads between formulation science and clinical translation. Novasomes are an interesting type of nanovesicle for local drug delivery in cutaneous oncology due to their high drug-loading capacity, tunable release, skin penetration supporting properties. They offer a modular design and can be used for precision therapy in the fields of topical and intralesional application, where local efficacy and reduced systemic toxicity are desired. Looking to the future, concerted consortia, measurement of validated in vitro, in vivo, and in vitro correlations, and the development of early phase imaging led clinical trials will be necessary to achieve the clinical potential of novasomes and ensure incorporation of real-time dosimetry in the routine care of cutaneous cancer. This review concentrates on the interplay among formulation engineering, theranostic monitoring, dosing optimization, and the translation to the clinic, rather than just on the aspect of carrier design or therapeutic performance, to present a bigger picture for the future clinical implementation.

To sum up, novasomes are a multifunctional nanovesicular system with many promises in targeted drug delivery, controlled drug release and in vivo monitoring and personalized dosage in cutaneous oncology, but many of the advantages that have been proposed so far are still largely based on preclinical findings, and direct comparisons with clinically approved nanocarriers are limited. Standardization of characterization techniques, thorough translational studies and well-designed clinical trials are necessary to prove clinically relevant therapeutic benefits in actual oncology settings.

## Data Availability

No datasets were generated or analysed during the current study.
